# The associations of tobacco use, sexually transmitted infections, HPV vaccination, and screening with the global incidence of cervical cancer: an ecological time series modeling study

**DOI:** 10.4178/epih.e2023005

**Published:** 2022-12-13

**Authors:** Luyan Zheng, Yushi Lin, Jie Wu, Min Zheng

**Affiliations:** State Key Laboratory for Diagnosis and Treatment of Infectious Diseases, National Clinical Research Center for Infectious Diseases, Collaborative Innovation Center for Diagnosis and Treatment of Infectious Diseases, The First Affiliated Hospital, College of Medicine, Zhejiang University, Hangzhou, China

**Keywords:** Human papillomavirus, Cervical cancer, Incidence, Modeling, Global

## Abstract

**OBJECTIVES:**

We aimed to quantify the temporal associations between cervical cancer incidence and cervical cancer-related factors and to predict the number of new cervical cancer cases averted under counterfactual scenarios compared to the status quo scenario.

**METHODS:**

We described temporal trends in cervical cancer and associated factors globally from 1990 to 2019. We then used generalized linear mixed models to explore the impact of tobacco use, sexually transmitted infections (STIs), human papillomavirus (HPV) vaccination, and cervical screening on cervical cancer incidence. A counterfactual analysis was performed to simulate the most effective scenario for reducing cervical cancer incidence.

**RESULTS:**

The worldwide incidence of cervical cancer showed a downward trend over the past 3 decades (estimated annual percentage change, -0.72%), although the incidence remained high (>30 cases per 100,000 persons) in sub-Saharan Africa, Latin America, and the Caribbean. Higher smoking and STI prevalence showed significant direct associations with the incidence of cervical cancer, whereas HPV vaccination and screening coverage showed significant inverse associations. If the strategic goals for accelerating the elimination of cervical cancer and tobacco control programs had been achieved in 2019, the largest decrease in the number of new cervical cancer cases would have been observed, with 54,169 fewer new cases of cervical cancer in 2019.

**CONCLUSIONS:**

Our counterfactual analysis found that a comprehensive intervention program emphasizing scaled-up cervical screening coverage (70%), HPV vaccination coverage (90%), and tobacco control (30% relative reduction) would be the most effective program for reducing cervical cancer incidence.

## INTRODUCTION

Cervical cancer is a reproductive system cancer arising from infection via high-risk types of human papillomavirus (HPV) and advancing to cervical intraepithelial neoplasia (CIN), culminating in malignancy initiation [1]. According to the Global Cancer Statistics in 2020, cervical cancer was the fourth most frequently diagnosed cancer in women [2], with an estimated 604,000 new cases and 342,000 deaths worldwide. It is also the most common cause of cancer-related mortality in women in developing countries, and nearly 9 women in 10 women who die from cervical cancer live in low-income and middle-income countries (LMICs) [3]. Premature death from cervical cancer is a preventable tragedy for hundreds of thousands of women and their families each year [4]. Several strategies have been proposed to prevent the onset and progression of cervical cancer, including HPV vaccination, cervical screening, and treatment of high-grade pre-cancer, which all show high effectiveness [5,6].

Although an estimated 80% of women will experience at least 1 genital HPV infection during their lifetime, up to 60% of genital HPV infection-caused CIN cases regress spontaneously, and fewer than 1% of CIN cases eventually progress to cervical cancer [7]. Smoking and sexually transmitted infections (STIs), especially human immunodeficiency virus (HIV), herpes simplex virus-2 (HSV-2), and syphilis infections, are synergistic exposures that increase the risk of eventual progression to carcinogenesis [8,9]. Smoking causes cervical cancer through somatic mutations and DNA methylation, while many sexually transmitted pathogens (except HPV) have an indirect role in oncogenesis, mainly by suppressing a woman’s T helper, cell-mediated immune response, thereby enhancing the effects of high-risk HPV [9].

Over the past 30 years, much effort has been focused on eliminating cervical cancer. In the 1990s, cervical cytology screening was proposed for early cancer detection, and the first HPV vaccine was officially launched in 2006 [10]. Currently, cervical cancer is one of the cancers that can be effectively prevented by vaccination and screening. In November 2020, the World Health Organization (WHO) launched the Global Strategy for Accelerating the Elimination of Cervical Cancer [11]. However, the implementation of HPV vaccination and screening and the prevalence of smoking and STIs vary considerably among different countries, which poses enormous challenges for achieving the goals of the global strategy to accelerate the elimination of cervical cancer.

Given the different stages of cervical cancer incidence and its risk factors across countries, consolidating the evidence of ecological associations between cervical cancer incidence and cervical cancer-related factors is essential for further intervention plans. In addition, few studies have predicted the impacts of varying levels of cancer-related factors on cervical cancer incidence at the global level under counterfactual scenarios. In this study, we aimed to (1) quantify the temporal associations between cervical cancer incidence and cervical cancer-related factors using observations from 204 countries and territories from 1990 to 2019, and (2) predict the number of new cervical cancer cases that would be averted under counterfactual scenarios compared to the status quo scenario.

## MATERIALS AND METHODS

### Study design

We carried out an ecological time series analysis to explore whether changes in 4 key factors produced any trends in cervical cancer across 204 countries and territories across the globe. We collected longitudinal data on smoking prevalence, STI prevalence, HPV vaccination rates, and cervical cancer screening coverage at the national level in 204 countries. We examined the ecological associations between the above 4 risk/protective factors and the incidence rates of cervical cancer at the global level.

### Data sources

#### Cervical cancer outcome data

The dependent variable was composed of longitudinal, country-specific, age-standardized estimates of cervical cancer incidence. Annual estimates for incidence due to cervical cancer by age and gender for 204 countries and territories from 1990 to 2019 are available from the Global Burden of Disease (GBD) Study 2019 [12]. The GBD study is a scientific effort to quantify the comparative magnitude of health loss due to disease, injury, and risk factors by age, gender, and geography for specific points in time. The details on the estimation process for the disease burden of cervical cancer have been published elsewhere [12,13]; herein, we briefly describe the estimation process. First, the major input data for incidence estimates were obtained from 3,303 cervical cancer registries globally, which were systematically identified and extracted to determine cervical cancer incidence. The input data sources for cervical cancer estimates are available at http://ghdx.healthdata.org/gbd-2019/data-input-sources?. Second, since coding systems were inconsistent among cancer registries for various reasons, different coding systems from the reference definition in the International Classification of Diseases, 10th revision were used to adjust for this bias. Finally, cervical cancer incidence was estimated using DisMod-MR, version 2.1 (WHO), which is a Bayesian meta-regression tool that allows the evaluation of all available data on incidence, prevalence, remission, and mortality for a disease, thereby ensuring consistency between epidemiological parameters [12].

#### Data on risk factors

Although many factors contribute to the progression from HPV infection to cervical cancer, such as intrauterine device usage and a diet low in fruits and vegetables, most of them have only been reported in individual-level studies [14-16]. Given the accuracy, reliability, and completeness of the risk factor data, we used tobacco use and STI prevalence data of women in the current study. Estimates of the prevalence of tobacco use and the number of people who currently use smoked tobacco products are available from the GBD Study [17]. Tobacco use prevalence is measured as the percentage of the population (≥ 15 years) who currently smoke any tobacco product (excluding the use of smokeless tobacco), whether on a daily or non-daily basis, in a year. These data are presented by gender, age group, and year for 204 countries and territories for 1990 to 2019. Country-specific and age-standardized prevalence of STIs for 204 countries and territories between 1990 and 2019 are also available from the GBD Study [12]. The STIs reported in the GBD study include HIV/AIDS, syphilis, chlamydial infection, trichomoniasis, genital herpes, and other STIs. The full details on the estimation process for tobacco use prevalence and STI prevalence have been published elsewhere [12].

#### Data on preventive factors

We adopted the data representing official cervical screening coverage from the WHO [18]. Cervical cancer screening coverage was defined as follows: countries indicating that they had a national cervical cancer screening program were asked to indicate the coverage of the program, as less than 10%, 10% to 50%, more than 50% but less than 70%, or 70% or more. Data on screening coverage were available only for 2015, 2017, and 2019, and missing data were observed in 38 out of 150 countries. Moreover, estimates of national immunization coverage are reported annually through the WHO-United Nations Children Fund (UNICEF), which includes the first dose of official HPV vaccination coverage among the target population for each reporting year from 2010 to 2019 [19]. However, country-specific HPV vaccination rates were discontinuous during that time. Among 98 countries with national HPV vaccination programs, 17 had missing data on HPV vaccination coverage. Briefly, we imputed missing values with given values, nearest neighbor imputation, or linear interpolation. Summary information on the missing data and imputation methodology can be found in Supplementary Materials 1 and 2 (section 1).

### Statistical analysis

#### Descriptive analysis

We first conducted a descriptive analysis of cervical cancer incidence and the 4 key factors. To graphically demonstrate the distribution of cervical cancer and its risk factors across the globe in 2019, we generated several choropleth maps of the age-standardized rates (ASRs) of cervical cancer, tobacco use, and STI prevalence rates. We then computed the estimated annual percentage change (EAPC), which is a summary and widely used measure of the ASR trend over a specified interval [20,21], to illustrate temporal trends and variations between countries in the ASR of cervical cancer, tobacco use, and STI prevalence rates in selected countries from 1990 to 2019. Next, we calculated the absolute change in the number of women with cervical cancer, tobacco use, and STIs from 1990 to 2019. In addition, to graphically demonstrate temporal trends in preventive factors of cervical cancer, we plotted the cervical screening rates and HPV vaccination rates using both maps and stacked bars.

#### Model development

We introduced 2-year lagged cervical screening coverage and 5-year lagged tobacco use prevalence, STI prevalence, and HPV vaccination rates into the model to account for time-dependent effects; the details are available in the Supplementary Material 2 (section 2). We used a generalized linear mixed (GLM) model fitted by restricted maximum likelihood to assess the time series associations between tobacco use, STIs, HPV vaccination rate, and screening coverage with the incidence of cervical cancer. GLM models can be viewed as an extension of linear mixed models to allow for response variables from different distributions, such as binary responses, or an extension of generalized linear models to include both fixed and random effects (hence “mixed” models) [22]. We constructed a total of 3 GLM models and assessed model fit using the Akaike information criterion (AIC), where a smaller AIC is preferred [23]. Further details on the model-selection procedure are provided in the Supplementary Material 2 (section 3). Our final model took the following form in the present study:

log(*y_c,t_*)=*β_0_*+*β_s_*S_*c,t*_+*β_v_*log(V_*c,t*_)+*β_t_*log(*T_c,t_*)+β_*i*_log(*I_c,t_*)+a_c_+ε_*c,t*_

where *y_c,t_* is the incidence of cervical cancer in each country (c) and year (*t*), *β_0_* is the intercept for the model, and *β_s_*, *β_v_*, *β_t_*, and *β_i_* denote the fixed effects for screening coverage, HPV vaccination coverage, tobacco use prevalence, and STI prevalence, respectively. *S_c,t_, V_c,t_, T_c,t_, and I_c,t_* refer to the screening coverage, HPV vaccination coverage, tobacco use prevalence, and STI prevalence for country *c* in year *t*. Finally, *a_c_* is the random intercept for country *c*. We conducted all analyses with RStudio version 1.4.1103 (R Foundation for Statistical Computing, Vienna, Austria) using the R packages lme4 (version 1.1.27.1) and lmerTest (version 3.1.3).

### Counterfactual analysis

To further explore and quantify the impact of tobacco use, STIs, HPV vaccination, and screening coverage on global cervical cancer incidence, we modeled the incidence of cervical cancer in 2019 in all countries under 5 simulated scenarios, which were based on the WHO’s Global Strategy for Accelerating the Elimination of Cervical Cancer [24] and the Tobacco Control Program (TCP) (2019-2025) [25]. For the former, the main task is to achieve 90% HPV vaccination coverage and 70% screening coverage in all countries by 2030, while the latter aims to achieve a 30% relative reduction in tobacco use among people aged 15 and older compared to current rates in 2025. The characteristics and parameters of the simulated scenarios are shown in Supplementary Material 3.

We used random effects in model fitting, but not in the counterfactual analysis. We calculated simulated incidence rates by multiplying the estimated marginal effect of each factor by the alternative values proposed in each of the counterfactual scenarios for each country-year. We computed the global population-weighted average for the status quo and simulated scenarios using population data sourced from the GBD Study of 2019. Next, we calculated the number of new cervical cancer cases in each scenario based on the predicted incidence rates and population data. Finally, the additional reduction in the number of new patients was obtained by subtraction between the status quo and simulated scenarios. We employed the 2.5% and 97.5% quantiles from 1,000 draws (unbiased random samples) of the uncertainty distribution of each of the estimated coefficients to generate 95% uncertainty intervals (UIs).

### Ethics statement

Additional ethical approval is not required because all analyses were conducted using publicly available data without personal identification information.

## RESULTS

**Figure 1.**
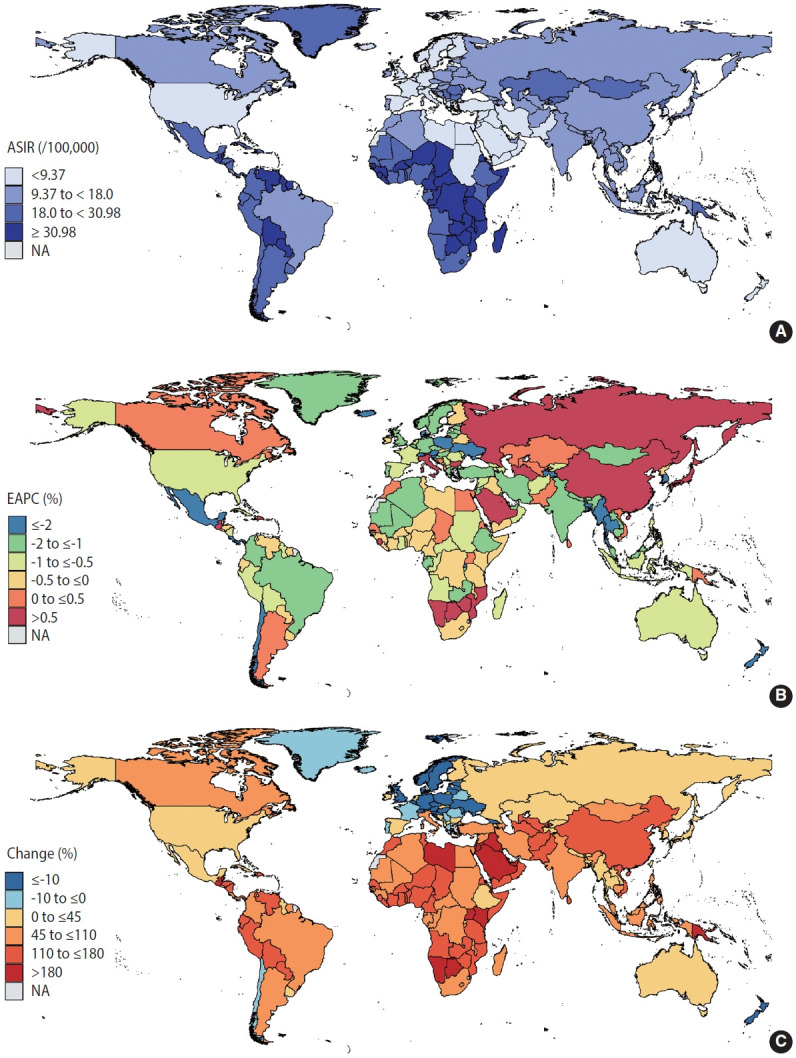
Choropleth maps showing geographic variation in ASIR of cervical cancer. (A) The ASIR per 100,000 persons in 2019, (B) the EAPC of the ASIR between 1990 and 2019, (C) changes in incident cases between 1990 and 2019. ASIR, age-standardized incidence rate; EAPC, estimated annual percent change; NA, nat available.

According to the GBD Study, the number of new cervical cancer cases rose from 338,450 in 1990 to 565,541 in 2019. A total of 2.89 million patients with cervical cancer were reported to have cervical cancer in 2019. Figure 1A provides a global profile of the age-standardized incidence rates (ASIRs) for cervical cancer in 2019. The ASIR exceeded 30 cases per 100,000 women in sub-Saharan Africa, Latin America and the Caribbean, mostly in LMICs. Incidence rates below 9 cases per 100,000 persons were seen in high-income North America, Australasia, and some countries in North Africa and the Middle East. From 1990 to 2019, the global ASIRs of cervical cancer decreased by an average of 0.72% annually, but the incidence rates in 38 countries increased (Supplementary Material 4). Figure 1B shows that a continuous rise in the incidence of cervical cancer was mainly observed in East Asia and Eastern Europe (EAPC > 0.5%). Declining cervical cancer incidence trends were primarily noted in South Asia, Southeast Asia, Australasia, Latin America, and some countries in Africa (EAPC < -0.5%). Compared to 1990, the absolute number of people with cervical cancer is increasing in almost all countries in the world in 2019 (Figure 1C). The absolute number of changes in cervical cancer has doubled over the past 3 decades in Southeast Asia, sub-Saharan Africa, Latin America, and some countries in North Africa and the Middle East.

**Figure 2.**
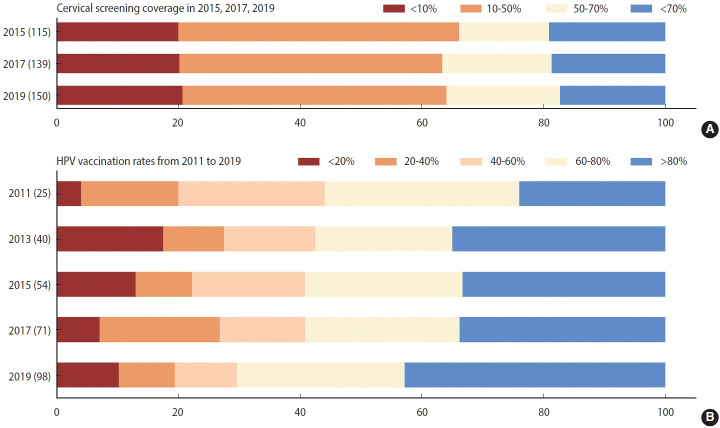
Level of coverage of cervical screening and human papillomavirus (HPV) vaccination. (A) Cervical screening coverage in 115, 139, and 150 countries in 2015, 2017, and 2019 respectively, (B) HPV vaccination rates in 98 countries from 2011 to 2019.

Like the burden of cervical cancer, the distribution of its risk and protective factors varied across regions. First, the age-standardized prevalence of tobacco use was notably higher (> 18%) in Southern Latin America and Western Europe than in the other regions in 2019 (Supplementary Material 5). Despite comparatively low tobacco use prevalence (< 7%), the absolute number of smokers has doubled over the past three decades in LMICs in Africa. Supplementary Material 6 indicates that STI prevalence rates were significantly higher in LMICs in sub-Saharan Africa, Latin America, and the Caribbean (> 33,832 cases per 100,000 persons) than in other regions. STI prevalence below 16,134 per 100,000 people was seen in high-income North America, Australasia, and South Asia. Supplementary Material 7 reveals that countries with high screening coverage mainly consisted of high-income North America, Tropical Latin America, some European countries, and 3 countries in Asia (India, South Korea, and Kazakhstan). Fourth, we found that the HPV vaccine was introduced in increasingly many countries between 2010 and 2019, and approximately half of countries worldwide were able to reach more than 80% coverage of HPV vaccination as of 2019 (Figure 2). High vaccination rates were primarily noted in middle-social development index (SDI), high-middle–SDI, and high-SDI nations (Supplementary Material 8).

Our adjusted analysis indicated that higher tobacco use and STI prevalence were significantly associated with increased incidence rates of cervical cancer between 1990 and 2019, but higher cervical screening coverage and HPV vaccination rates were significantly associated with decreased incidence rates of cervical cancer (Table 1).

Based on the prespecified counterfactual scenarios, we derived the following results. If cervical screening and HPV vaccination programs had not been introduced in any country in 2019, we estimate that the cervical cancer incidence would have been higher than the observed rates in 2019, with 28,646 more new cases of cervical cancer worldwide (Table 2). If the strategic goals set by the WHO’s Global Strategy for Accelerating the Elimination of Cervical Cancer (screening coverage up to 70% and HPV vaccination rates up to 90% for all countries) had been achieved in 2019, we estimate that cervical cancer incidence in 2019 would have been lower than the observed values in 2019, with 43,394 fewer new cases of cervical cancer worldwide. If the strategic goals set by the TCP of the WHO (tobacco use prevalence declined by 30%) had been achieved in 2019, there would have been 11,427 fewer new cases of cervical cancer in 2019 than the observed number. If both goals set by the WHO had been achieved at the same time, the greatest progress in reducing cervical cancer incidence would have been observed, with approximately 54,169 fewer new cases of cervical cancer across all countries in 2019.

## DISCUSSION

We found that the worldwide incidence of cervical cancer showed a downward trend over the past 3 decades, although the incidence remained high (> 30 cases per 100,000 persons) in sub-Saharan Africa, Latin America and the Caribbean. Countries and regions with high cervical cancer incidence tend to have lower availability of screening and HPV vaccination, and higher tobacco use and STI prevalence. Moreover, we developed a simulation model of cervical cancer incidence, which also confirmed the ecological association between cervical cancer incidence and the above 4 factors at the population level. Further, our counterfactual analysis found that a comprehensive intervention program emphasizing scaled-up cervical screening would be the most effective program for reducing cervical cancer incidence.

Decreasing cervical cancer incidence trends were primarily seen in South Asia and in Central and Tropical Latin America. These regions also had upward trends in high cervical screening coverage and HPV vaccination rates from 2010 to 2019. Our model results confirm the effectiveness of cervical screening and HPV vaccination on cervical cancer incidence at the population level. Moreover, if the cervical screening coverage and HPV vaccination rate had reached 90% and 70% for 204 countries and territories globally, there would have been 26,266 and 18,076 fewer new cases of cervical cancer worldwide in women in 2019, respectively. The strong negative association between cervical cancer incidence rates and screening most likely reflects the key role of screening programs in reducing incidence by detecting cervical pre-cancer and cancer lesions early [26]. In the long term, however, HPV vaccination is the main strategy to realize the prospect of eliminating cervical cancer.

Regrettably, 20% of countries worldwide still had not introduced national cervical screening programs, and 50% of countries worldwide still had not introduced national HPV vaccination programs as of 2019, mostly in LMICs. There are considerable disparities in cervical screening coverage and HPV vaccination rates among countries worldwide, an increasingly problematic issue that could hinder global efforts to eradicate cervical cancer [27]. A lack of perceived risk of HPV infection and high costs are among the most commonly reported reasons for screening nonattendance and HPV vaccine hesitance in LMICs [28,29]. To improve HPV risk perceptions, obstetricians and gynecologists are potential entry points for women of childbearing age. Women who obtain information about cervical cancer from health professionals are 6 times more likely to agree to undergo screening [30]. In economic terms, international donors can be an important source in developing countries, similar to the Global Alliance for Vaccines and Immunization [31], which provided a record low price of as little as US$4.50 per dose for low-income countries as early as 2013 [29].

Countries and regions with high cervical cancer incidence were primarily located in sub-Saharan Africa and Latin America (except southern Latin America). These regions also have very high levels of STIs compared to the rest of the world. Although most STIs are not usually fatal, they can increase the infectiousness of and susceptibility to HPV. The WHO estimated that nearly 1 million people become infected with curable STIs every day [32], with nearly more than 90% of STIs occurring in LMICs [33]. Indeed, several different strategies for STI management and control have been proposed, such as syndromic management, presumptive periodic treatment, and partner notification [32]. Among these, point-of-care tests have crucial implications for STI control in LMICs due to their affordability, sensitivity, and specificity [34]. Given their large population size and poor affordability, policymakers should also prioritize controlling STIs in LMICs when developing strategies to reduce the disease burden caused by cervical cancer.

In addition, a positive association between tobacco use prevalence and cervical cancer incidence among women was also observed in our study, which was consistent with previous findings [35]. In fact, tobacco smoke, is a complex mixture of chemicals among which at least 60 are carcinogens [36], and it is associated with at least 17 types of human cancer, including the development of cervical cancer from an HPV infection [37]. Although tobacco use prevalence among women has decreased from 17% in 2000 to 10% in 2015 [38], the prevalence of cigarette smoking among adult women was still as high as 13% in 2016 and there was no decrease from 2016 to 2019 [39]. As smoking increases the risk of cervical cancer, appropriate interventions must be taken to prevent smoking in order to accelerate the elimination of cervical cancer. Previous studies have demonstrated that stress is the most frequently reported reason for the onset of smoking among women, and many non-pharmacological interventions have proven to be effective against smoking, such as psychological consultation and community-based education programs [40,41].

### Strengths and limitations

Our study is the first to provide quantitative estimates of the additional health benefits that would be realized if counterfactual scenarios for cervical cancer control are achieved. Our study also has some limitations. First, causality cannot be inferred from this ecological time series study due to the use of aggregated data and the risk of the ecological fallacy. Second, imputation for missing values of HPV vaccination and coverage of cervical cancer screening, especially in developing countries, may overestimate the effect of HPV vaccine and cancer screening. However, we deliberately imputed those values conservatively, which may somewhat offset this overestimation. Third, another limitation of the current study relates to the lack of data on confounding factors, which may not fully explain the trends of cervical cancer incidence. We included only 4 major factors, while other factors that are significantly associated with cervical cancer (e.g., education, high fertility, and intercourse during menstruation) were not included due to the unavailability of data. Fourth, measures for tobacco use, STIs, and HPV vaccination required a longer period of time to be implemented effectively. However, 5-year lag data were used in the model because of the limited time range of our data, which might have resulted in overestimating their effect. Last but not least, data used for the model were collected from multiple databases, and the quality of cancer registry data may have varied over a long period of time. Although we employed Bayesian statistics to account for the inconsistencies of data, large variance may exacerbate uncertainties in the effect estimates.

In conclusion, worldwide cervical cancer incidence has declined over the past 3 decades, which was associated with expanded cervical screening and HPV vaccination programs. However, there are still substantial disparities in cervical cancer incidence among countries, mostly in LMICs. Given their large population size and potential for greater health benefits, LMICs deserve more attention and supportive policies in expanding cervical screening and HPV vaccination programs, while public health interventions are urgently needed to address high-prevalence risk factors, such as tobacco use and STIs.

## DATA AVAILABILITY

The data on cervical cancer incidence, tobacco use prevalence, STI prevalence, cervical screening coverage and HPV vaccination rates used in these analyses are available online and from the authors on request (cervical cancer incidence, tobacco use prevalence, and STI prevalence: http://ghdx.healthdata.org/gbd-2019/data-input-sources?; cervical screening coverage: https://apps.who.int/gho/data/view.main.UHCCERVICALCANCERv; HPV vaccination rates: https://immunizationdata.who.int/pages/coverage/hpv.html?GROUP=Countries+WHO%20Regions&CODE=Global&YEAR=.).

## Figures and Tables

**Table 1.** Percentage changes in current cervical cancer incidence rates based on fixed effect coefficients from adjusted mixed effect linear regression models

Factors^1^	Relative change (%)	95% UI		p-value
		LL	UL	
Cervical screening	-2.442	-2.920	-1.975	<0.001
HPV vaccination	-0.278	-0.403	-0.161	<0.001
Tobacco use	6.484	4.725	8.388	<0.001
STIs	12.402	9.346	15.282	<0.001

UI, uncertainty interval; LL, lower limit; UL, upper limit; HPV, human papillomavirus; STIs, sexually transmitted infections.

1 Cervical cancer incidence, HPV vaccination, tobacco use, and STI prevalence rates were log-transformed; Before log-transformation, tobacco use and the STI prevalence rates were normalized on a scale of 0-1.

**Table 2.** Simulated reduction in the global incidence rates of cervical cancer and the total number of patients with new-onset cervical cancer under counterfactual scenarios

Scenarios^1^	Relative change in incidence (%)	95% UI		Change in the no.	95% UI	
		LL	UL		LL	UL
Natural history	5.56	5.44	5.57	28,646	23,864	32,307
Improved cervical screening only	-5.10	-5.19	-5.05	-26,266	-29,249	-22,764
Improved HPV vaccination only	-3.51	-3.54	-3.27	-18,076	-18,971	-15,538
WHO cervical cancer target	-8.43	-8.56	-8.32	-43,394	-48,238	-37,531
WHO tobacco target	-2.28	-2.41	-1.97	-11,427	-11,764	-10,576
Ideal 1	-10.52	-10.64	-10.28	-54,169	-59,561	-46,652
Ideal 2	-8.45	-8.58	-8.35	-43,532	-48,388	-37,639

37,639UI, uncertainty interval; LL, lower limit; UL, upper limit; HPV, human papillomavirus; WHO, World Health Organization; TCP, Tobacco Control Program.

1 The 7 scenarios were as follows: (1) Natural history: we simulated no cervical screening or HPV vaccination programs in 2019; (2) Improved cervical screening only: we simulated that the strategic goal for cervical screening launched by the Cervical Cancer Screening Program of the WHO was achieved in 2019; (3) Improved HPV vaccination only: we simulated that the strategic goal for HPV vaccination rates launched by the Cervical Cancer Screening Program of the WHO was achieved in 2019; (4) WHO cervical cancer target: we simulated that the strategic goals for cervical screening and HPV vaccination launched by the Cervical Cancer Screening Program of the WHO were achieved in 2019; (5) WHO tobacco target: we simulated that the strategic goals launched by the TCP (2019-2025) of the WHO were achieved in 2019; (6) Ideal 1: we simulated that the strategic goals set by the Cervical Cancer Screening Program and TCP of WHO were achieved in 2019 at the same time; (7) Ideal 2: we simulated that both cervical screening coverage and HPV vaccination rates increased to 100%.
