# Supplementary material for: The associations of tobacco use, sexually transmitted infections, HPV vaccination, and screening with the global incidence of cervical cancer: an ecological time series modeling study
Source: Epidemiol Health. 2022 Dec 13;45:e2023005. doi: 10.4178/epih.e2023005 (PMC10581889; doi:10.4178/epih.e2023005)
Supplement: Supplementary file 1 [file epih-45-e2023005-Supplementary.docx]

**Supplementary Material**

**The associations of tobacco use, sexually transmitted infections, HPV vaccination, and screening with the global incidence of cervical cancer:**

**An ecological time series modelling study**

**Contents**

[Section 1. Methodology of imputation with missing data 2](#_Toc103065375)

[Section 2. Methodology of data preprocessing 3](#_Toc103065376)

[Section 3. Methodology of model selection. 4](#_Toc103065377)

[Section 4. Global distribution of preventive factors of cervical cancer. 5](#_Toc103065378)

[eFigure 1. Choropleth maps showing geographic variation in age-standardized prevalence rate of tobacco use. 5](#_Toc103065379)

[eFigure 2. Choropleth maps showing geographic variation in age-standardized prevalence rate of STIs. 6](#_Toc103065380)

[eFigure 3. Global cervical screening coverage from 115, 139 and 150 countries in 2015, 2017 and 2019. 7](#_Toc103065381)

[eFigure 4. Global HPV vaccination rates in 2015, 2017, 2019. 8](#_Toc103065382)

[Section 5. The estimation of changes to cervical cancer incidence. 9](#_Toc103065383)

[Table S1. Characteristics and parameters of simulated scenarios. 9](#_Toc103065384)

[Table S2. The estimated annual percent change of age-standardized incidence rate of cervical cancer in women between 1990 and 2019. 11](#_Toc103065385)

[References 17](#_Toc103065386)

# Supplementary Material 1: Section 1. Methodology of imputation with missing data

For cervical screening coverage, missing data were imputed with the following three steps. First, missing data between 2015 and 2019 were imputed with the given data from the previous year for the corresponding country. Second, since the World Health Organization (WHO) first introduced the “use human papillomavirus (HPV) tests to screen women for cervical cancer prevention” into the Comprehensive cervical cancer control-A guide to essential practice in 2006 [1], missing values before 2007 were imputed using “not available data”. Third, if the national cervical screening program was not introduced as of 2015, missing data between 2007 and 2014 for this country were imputed using “not available data”. Otherwise, missing data between 2007 and 2014 for this country were imputed using “less than 10%”.

For HPV vaccination rates, missing data were imputed with the following three steps. First, missing data between 2010 and 2019 were imputed with the given data from the previous year for the corresponding country. Second, since the first HPV vaccine became commercially available in 2006, missing values before 2007 were imputed using 0. Third, if the national HPV vaccination program was not introduced as of 2010, missing data between 2007 and 2009 for this country would be imputed using 0 because the estimated coverage of HPV vaccination was very low [2,3]. Otherwise, missing values between 2007 and 2009 for this country were replaced using linear interpolation.

# Supplementary Material 1: Section 2. Methodology of data preprocessing

Prior to modelling, the data were preprocessed through the following three steps. First, to ensure the same unit of magnitude, tobacco use and STIs prevalence were standardized between 0 and 1 using the following formula:

$$x=\frac{x-min}{max-min}$$

Second, all data except screening coverage were log transformed to satisfy the assumption of normality in the model. Third, log-transformed incidence rates of cervical cancer from 1990 to 2019 were matched to two-year lagged cervical screening scores and five-year lagged tobacco use prevalence, STIs prevalence, and HPV vaccination rates using country and year identifiers. The two-year lagged cervical screening coverage was introduced into the model due to its rapid onset of action, whereas three other factors were five-year lagged as they required a greater period of time to be implemented effectively. However, because of the limited time range of our data, introducing a longer lag period would have resulted in the loss of additional data points and further limiting our statistical power in detecting relevant associations between above three factors and cervical cancer incidence.

# Supplementary Material 1: Section 3. Methodology of model selection.

A series of models were developed to examine the associations between vaccination rates, screening coverage, tobacco use, STIs and cervical cancer incidence. Formulae used were as follows:

$$model 1: log \left( y_{c,t} \right)=\beta_{0}+\beta_{s}S_{c,t}+\beta_{v}\log\left( V_{c,t} \right)+\beta_{t}\log\left( T_{c,t} \right)+\beta_{i}\log\left( I_{c,t} \right) {+ a}_{c}{+ a}_{t}+ \varepsilon_{c,t}$$

$$model 2: log (y_{c,t})=\beta_{0}+\beta_{s}S_{c,t}+\beta_{v}\log(V_{c,t})+\beta_{t}\log(T_{c,t})+\beta_{i}\log(I_{c,t}) {+ a}_{c}+ \varepsilon_{c,t}$$

$$model 3: log (y_{c,t})=\beta_{0}+\beta_{s}S_{c,t} * \beta_{v}\log(V_{c,t})+\beta_{t}\log(T_{c,t})+\beta_{i}\log(I_{c,t}) {+ a}_{c}{+ a}_{t}+ \varepsilon_{c,t}$$

In model 1, we set above four factors as fixed factors and set the random intercept for the country (c) and year (t) at the same time. In model 2, we removed the random intercept for the year (t) based on model 1. In model 3, we consider the interaction effect between vaccination rates and screening coverage based on model 1. Model fit was assessed using the Akaike Information Criterion (AIC), where smaller AIC is preferred [4]. The main model applied to the following counterfactual analysis was model 2, which was chosen because its AIC values was low and was more directly interpretable.

# Section 4. Global distribution of preventive factors of cervical cancer.

## Supplementary Material 5. Choropleth maps showing geographic variation in age-standardized prevalence rate of tobacco use.


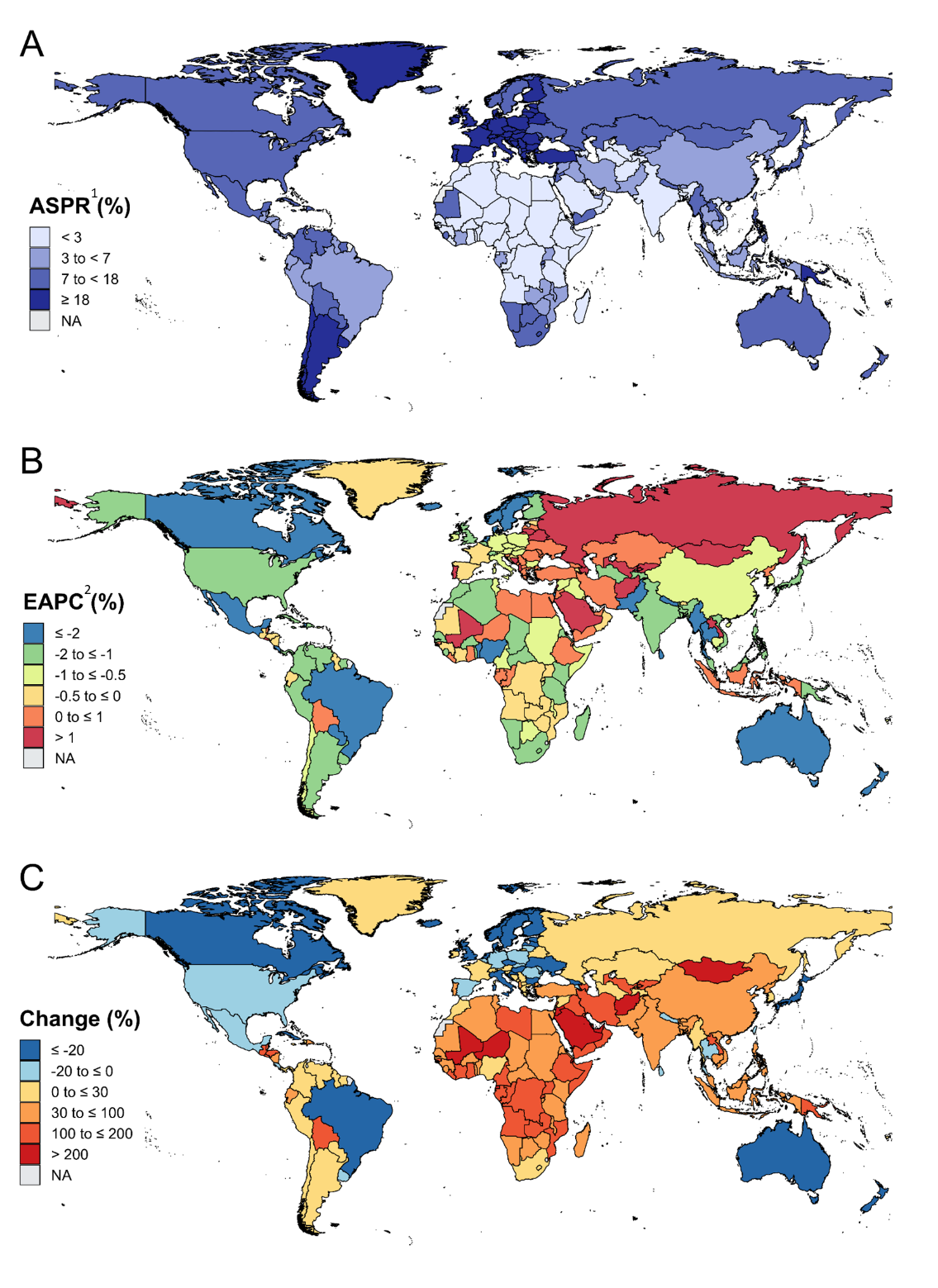


^1^ ASPR, age-standardized prevalence rate

^2^ EAPC, estimated annual percent change

A for age-standardized prevalence rate per 100,000 persons in 2019; B for EAPC of age-standardized prevalence rate between 1990 and 2019; C for change of prevalent cases in 1990 and in 2019;

## Supplementary Material 6. Choropleth maps showing geographic variation in age-standardized prevalence rate of sexually transmitted infections.


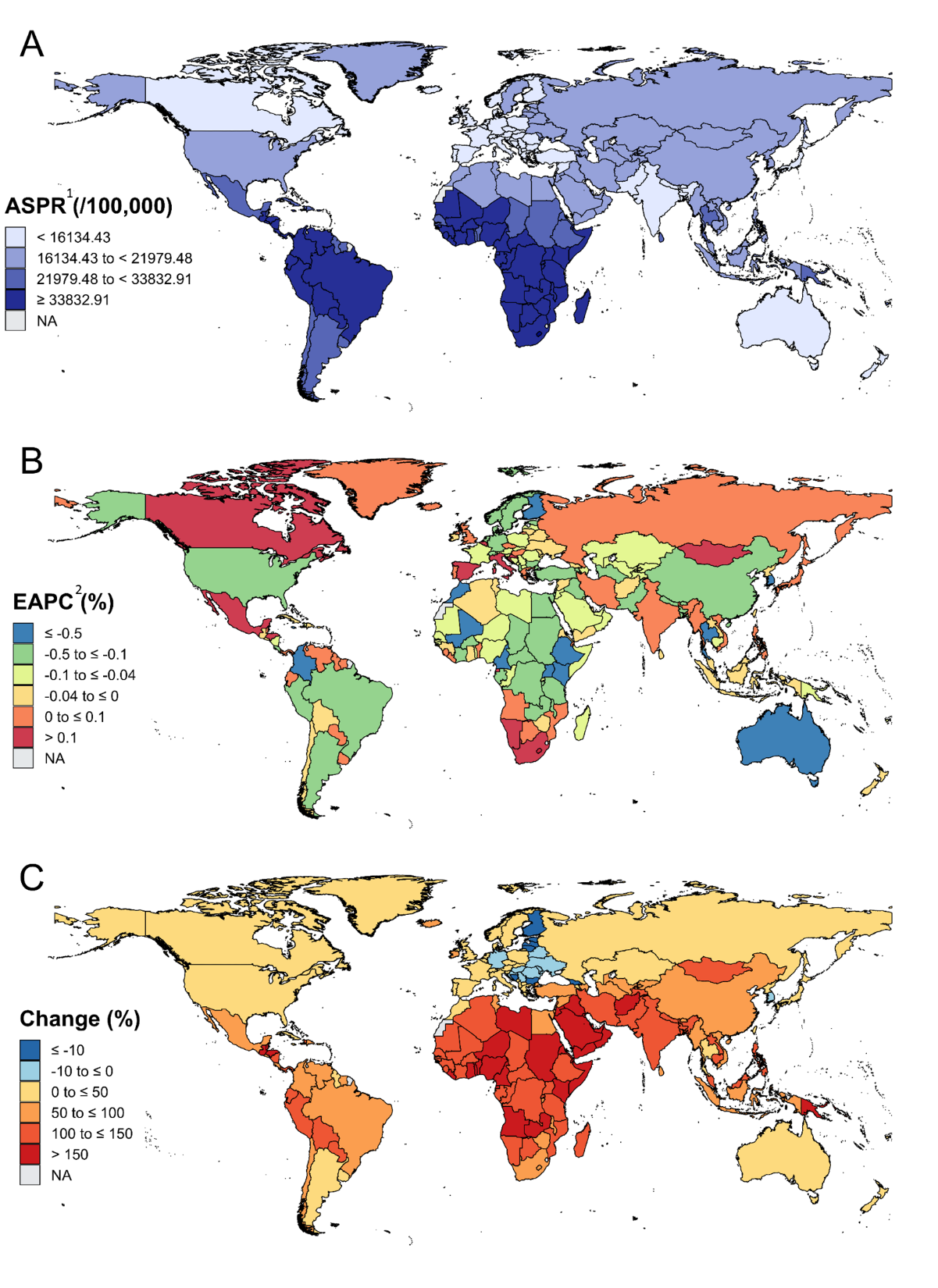


^1^ ASPR, age-standardized prevalence rate

^2^ EAPC, estimated annual percent change

A for age-standardized prevalence rate per 100,000 persons in 2019; B for EAPC of age-standardized prevalence rate between 1990 and 2019; C for change of prevalent cases in 1990 and in 2019;

## Supplementary Material 7. Global cervical screening coverage from 115, 139 and 150 countries in 2015, 2017 and 2019.


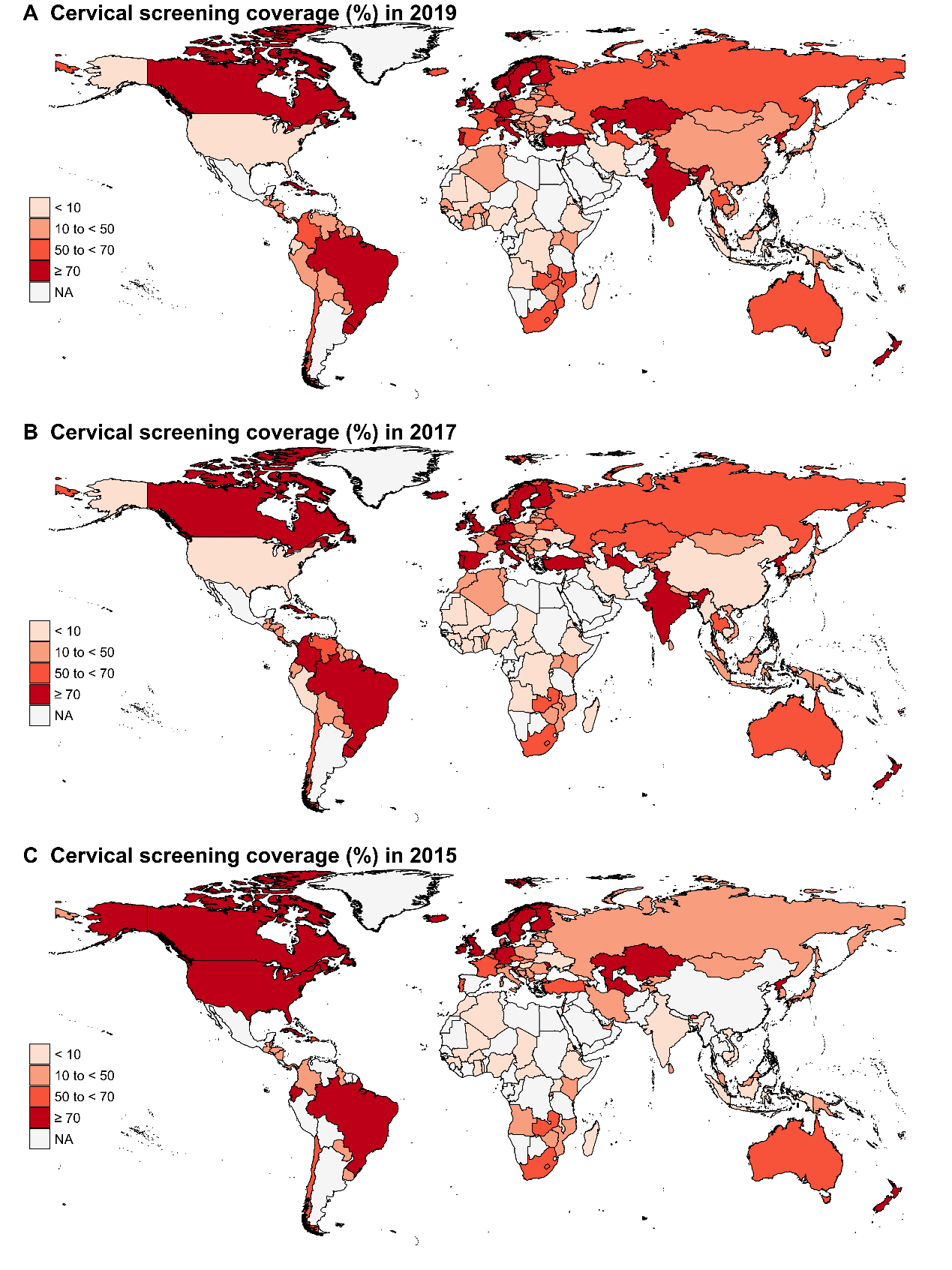


## Supplementary Material 8. Global HPV vaccination rates in 2015, 2017, 2019.


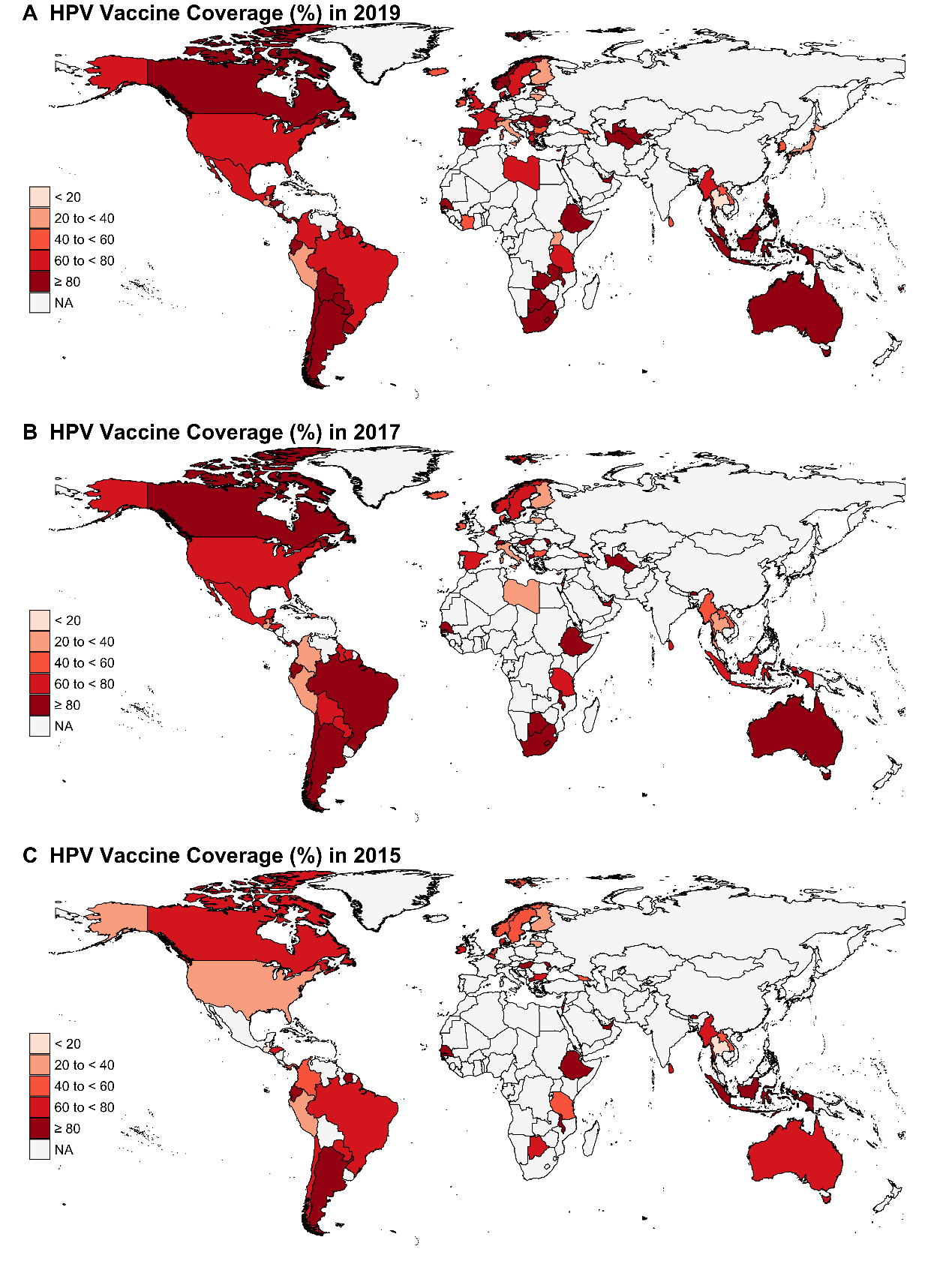


# Section 5. The estimation of changes to cervical cancer incidence.

## Supplementary Material 2. The percent of missing values for cervical screening and HPV vaccination coverage.

| Year | Cervical screening coverage | | |  | HPV^1^ vaccination coverage | | |
| --- | --- | --- | --- | --- | --- | --- | --- |
|  | No. of countries with available data | No. of countries with national screening program | Percent missing, % |  | No. of countries with available data | No. of countries with national vaccination program | Percent missing, % |
| 2010 | - | - | - |  | 18 | 19 | 5.26 |
| 2011 | - | - | - |  | 24 | 25 | 4.00 |
| 2012 | - | - | - |  | 31 | 32 | 3.13 |
| 2013 | - | - | - |  | 37 | 40 | 7.50 |
| 2014 | - | - | - |  | 46 | 48 | 4.17 |
| 2015 | 115 | 115 | 0.00 |  | 51 | 54 | 5.56 |
| 2016 | - | - | - |  | 60 | 63 | 4.76 |
| 2017 | 123 | 139 | 11.51 |  | 68 | 71 | 4.23 |
| 2018 | - | - | - |  | 80 | 80 | 0.00 |
| 2019 | 112 | 150 | 25.33 |  | 98 | 98 | 0.00 |

^1^ HPV, human papillomavirus

## Supplementary Material 3. Characteristics and parameters of simulated scenarios.

| **Scenarios** | **Factors** | **Parameters** |
| --- | --- | --- |
| Natural history | Cervical screening | No cervical screening program |
|  | HPV^1^ vaccination | 0 |
|  | Tobacco use | observed values in 2019 |
|  | STIs^2^ | observed values in 2019 |
| Improve cervical screening only | Cervical screening | 70% |
|  | HPV^1^ vaccination | observed values in 2019 |
|  | Tobacco use | observed values in 2019 |
|  | STIs^2^ | observed values in 2019 |
| Improve HPV vaccination only | Cervical screening | observed values in 2019 |
|  | HPV^1^ vaccination | 90% |
|  | Tobacco use | observed values in 2019 |
|  | STIs^2^ | observed values in 2019 |
| WHO^3^ cervical cancer target | Cervical screening | 70% |
|  | HPV^1^ vaccination | 90% |
|  | Tobacco use | observed values in 2019 |
|  | STIs^2^ | observed values in 2019 |
| WHO^3^ Tobacco target | Cervical screening | observed values in 2019 |
|  | HPV^1^ vaccination | observed values in 2019 |
|  | Tobacco use | reduced by 30% compared with observed values in 2019 |
|  | STIs^2^ | observed values in 2019 |
| Ideal 1 (achieved WHO^3^ cervical cancer target and WHO^3^ Tobaco target at the same time) | Cervical screening | 70% |
|  | HPV^1^ vaccination | 90% |
|  | Tobacco use | reduced by 30% compared with observed values in 2019 |
|  | STIs^2^ | observed values in 2019 |
| Ideal 2 (full prevention eligibility) | Cervical screening | 100% |
|  | HPV^1^ vaccination | 100% |
|  | Tobacco use | observed values in 2019 |
|  | STIs^2^ | observed values in 2019 |

^1^ HPV, human papillomavirus; ^2^ STIs, sexually transmitted infections; ^3^ WHO, World Health Organization

The seven scenarios were as follows: 1) Natural history: we simulated there was no cervical screening or HPV vaccination program in 2019. 2) Improve cervical screening only: we simulated that the strategic goal for cervical screening launched by the Cervical Cancer Screening Program of WHO were achieved in 2019. 3) Improve HPV vaccination only: we simulated that the strategic goal for HPV vaccination rates launched by the Cervical Cancer Screening Program of WHO were achieved in 2019. 4)WHO cervical cancer target: we simulated that the strategic goals for cervical screening and HPV vaccination launched by the Cervical Cancer Screening Program of WHO were achieved in 2019. 5) WHO Tobacco target: we simulated that the strategic goals launched by the Tobacco Control Program (2019-2025) (TCP) of WHO were achieved in 2019. 6) Ideal 1: we simulated that the strategic goals set by the Cervical Cancer Screening Program and TCP of WHO were achieved in 2019 at the same time. 7) Ideal 2: we simulated that both cervical screening coverage and HPV vaccination rates increased to 100%.

## Supplementary Material 4. The estimated annual percent change of age-standardized incidence rate of cervical cancer in women between 1990 and 2019.

| **Countries and territories** | **EAPC**^1^ **(%)** | **Upper** | **Lower** |
| --- | --- | --- | --- |
| Afghanistan | -0.51 | 0.66 | -1.66 |
| Albania | 0.23 | 1.7 | -1.23 |
| Algeria | -1.32 | -0.07 | -2.55 |
| American Samoa | 0.45 | 1.46 | -0.54 |
| Andorra | -0.34 | 0.82 | -1.47 |
| Angola | -0.79 | -0.08 | -1.49 |
| Antigua and Barbuda | -0.45 | 0.43 | -1.33 |
| Argentina | 0.38 | 1.19 | -0.42 |
| Armenia | -0.6 | 0.36 | -1.55 |
| Australia | -0.76 | 0.63 | -2.12 |
| Austria | -3.09 | -1.82 | -4.34 |
| Azerbaijan | -0.66 | 0.47 | -1.78 |
| Bahamas | -0.74 | 0.07 | -1.54 |
| Bahrain | -1.19 | 0.39 | -2.74 |
| Bangladesh | -2.2 | -1.04 | -3.36 |
| Barbados | -0.5 | 0.25 | -1.25 |
| Belarus | -0.5 | 0.51 | -1.5 |
| Belgium | -1.15 | 0.24 | -2.53 |
| Belize | -0.1 | 0.56 | -0.76 |
| Benin | -0.17 | 0.57 | -0.9 |
| Bermuda | -2.59 | -1.37 | -3.81 |
| Bhutan | -1.79 | -0.74 | -2.84 |
| Bolivia (Plurinational State of) | -0.79 | -0.17 | -1.4 |
| Bosnia and Herzegovina | 0.41 | 1.56 | -0.73 |
| Botswana | 0.74 | 1.36 | 0.13 |
| Brazil | -1.34 | -0.43 | -2.24 |
| Brunei Darussalam | -1.57 | -0.8 | -2.33 |
| Bulgaria | 1.56 | 2.51 | 0.62 |
| Burkina Faso | -0.26 | 0.46 | -0.97 |
| Burundi | -1.38 | -0.77 | -1.98 |
| Cabo Verde | -0.73 | 0.12 | -1.57 |
| Cambodia | -1.02 | -0.09 | -1.95 |
| Cameroon | -0.19 | 0.5 | -0.88 |
| Canada | 0.23 | 1.48 | -1.01 |
| Central African Republic | -0.54 | 0.06 | -1.15 |
| Chad | 0.34 | 1.04 | -0.36 |
| **Countries and territories** | **EAPC**^1^ **(%)** | **Upper** | **Lower** |
| Chile | -2.62 | -1.78 | -3.45 |
| China | 1.58 | 2.95 | 0.23 |
| Colombia | -1.6 | -0.75 | -2.45 |
| Comoros | -0.47 | 0.2 | -1.15 |
| Congo | -0.85 | -0.23 | -1.47 |
| Cook Islands | -0.8 | 0.56 | -2.14 |
| Costa Rica | -2.38 | -1.47 | -3.28 |
| Croatia | -1.68 | -0.63 | -2.72 |
| Cuba | -0.9 | -0.03 | -1.77 |
| Cyprus | -0.39 | 1.16 | -1.92 |
| Czechia | -1.87 | -0.8 | -2.93 |
| Côte d'Ivoire | -0.14 | 0.61 | -0.89 |
| Democratic People's Republic of Korea | -0.08 | 0.88 | -1.02 |
| Democratic Republic of the Congo | -0.34 | 0.36 | -1.04 |
| Denmark | -3.01 | -1.83 | -4.18 |
| Djibouti | -0.27 | 0.43 | -0.96 |
| Dominica | -0.85 | -0.18 | -1.52 |
| Dominican Republic | 0.92 | 1.78 | 0.07 |
| Ecuador | -0.06 | 0.72 | -0.83 |
| Egypt | 0.19 | 2.69 | -2.26 |
| El Salvador | -0.45 | 0.27 | -1.17 |
| Equatorial Guinea | -1.11 | -0.37 | -1.84 |
| Eritrea | 0.23 | 0.86 | -0.39 |
| Estonia | -1.41 | -0.48 | -2.32 |
| Eswatini | 1.06 | 1.7 | 0.43 |
| Ethiopia | -2 | -1.25 | -2.73 |
| Fiji | -0.1 | 0.56 | -0.75 |
| Finland | -0.1 | 1.69 | -1.85 |
| France | -0.95 | 0.41 | -2.29 |
| Gabon | -1.08 | -0.36 | -1.79 |
| Gambia | 0.36 | 1.21 | -0.48 |
| Georgia | -0.68 | 0.3 | -1.65 |
| Germany | -1.46 | -0.21 | -2.69 |
| Ghana | -0.96 | -0.23 | -1.68 |
| Global | -0.38 | 0.73 | -1.47 |
| Greece | -1.13 | 0.25 | -2.48 |
| Greenland | -1.73 | -1 | -2.46 |
| Grenada | -0.68 | 0.01 | -1.36 |
| Guam | -0.78 | 0.35 | -1.89 |
| Guatemala | 1.04 | 1.78 | 0.3 |
| Guinea | -0.23 | 0.32 | -0.78 |
| Guinea-Bissau | -0.03 | 0.57 | -0.63 |
| **Countries and territories** | **EAPC**^1^ **(%)** | **Upper** | **Lower** |
| Guyana | -0.73 | -0.11 | -1.35 |
| Haiti | -0.83 | -0.24 | -1.4 |
| Honduras | -0.24 | 0.69 | -1.16 |
| Hungary | -1.79 | -0.81 | -2.76 |
| Iceland | -2.41 | -0.92 | -3.88 |
| India | -1.09 | 0.01 | -2.17 |
| Indonesia | -0.68 | 0.39 | -1.75 |
| Iran (Islamic Republic of) | -1.31 | 0.65 | -3.24 |
| Iraq | -0.03 | 1.92 | -1.94 |
| Ireland | -0.1 | 1.17 | -1.36 |
| Israel | -0.03 | 1.55 | -1.58 |
| Italy | 1.95 | 3.6 | 0.33 |
| Jamaica | 0.2 | 0.93 | -0.51 |
| Japan | 0.8 | 2.08 | -0.47 |
| Jordan | -1.63 | 0.22 | -3.44 |
| Kazakhstan | 0.07 | 0.99 | -0.85 |
| Kenya | -0.16 | 0.78 | -1.1 |
| Kiribati | -0.61 | -0.23 | -0.99 |
| Kuwait | -1.54 | 0.35 | -3.39 |
| Kyrgyzstan | -0.75 | 0.16 | -1.65 |
| Lao People's Democratic Republic | -1.95 | -1.03 | -2.86 |
| Latvia | -1.66 | -0.43 | -2.87 |
| Lebanon | -0.74 | 0.88 | -2.35 |
| Lesotho | 3.3 | 3.98 | 2.62 |
| Liberia | -0.38 | 0.33 | -1.09 |
| Libya | -0.11 | 1.35 | -1.54 |
| Lithuania | -1.32 | -0.3 | -2.33 |
| Luxembourg | -2.26 | -0.75 | -3.74 |
| Madagascar | -0.7 | -0.02 | -1.38 |
| Malawi | -0.45 | 0.17 | -1.07 |
| Malaysia | -1.12 | -0.15 | -2.08 |
| Maldives | -3.85 | -2.71 | -4.96 |
| Mali | -1.03 | -0.3 | -1.76 |
| Malta | -1.06 | 0.7 | -2.78 |
| Marshall Islands | 0.03 | 0.69 | -0.63 |
| Mauritania | -1.06 | -0.34 | -1.77 |
| Mauritius | -2.09 | -1.04 | -3.13 |
| Mexico | -2.81 | -1.99 | -3.62 |
| Micronesia (Federated States of) | -0.3 | 0.38 | -0.98 |
| Monaco | -0.8 | 0.54 | -2.12 |
| Mongolia | -1.52 | -0.72 | -2.32 |
| Montenegro | -0.04 | 1.04 | -1.1 |
| **Countries and territories** | **EAPC** ^1^ **(%)** | **Upper** | **Lower** |
| Morocco | 0.05 | 1.14 | -1.03 |
| Mozambique | 0.58 | 1.25 | -0.09 |
| Myanmar | -2.1 | -1.17 | -3.02 |
| Namibia | 1.16 | 2.02 | 0.31 |
| Nauru | -0.3 | 0.33 | -0.92 |
| Nepal | -1.84 | -0.8 | -2.88 |
| Netherlands | -0.71 | 0.8 | -2.21 |
| New Zealand | -2.86 | -1.42 | -4.28 |
| Nicaragua | -0.8 | -0.09 | -1.49 |
| Niger | -0.26 | 0.45 | -0.95 |
| Nigeria | -0.19 | 0.75 | -1.12 |
| Niue | -0.66 | 0.13 | -1.44 |
| North Macedonia | -0.62 | 0.39 | -1.62 |
| Northern Mariana Islands | -0.9 | -0.25 | -1.56 |
| Norway | -1.58 | -0.28 | -2.86 |
| Oman | -0.59 | 0.91 | -2.07 |
| Pakistan | 0.08 | 1.55 | -1.37 |
| Palau | -0.54 | -0.05 | -1.03 |
| Palestine | -0.94 | 0.99 | -2.82 |
| Panama | -2.03 | -1.26 | -2.8 |
| Papua New Guinea | 0.31 | 1.18 | -0.55 |
| Paraguay | -0.22 | 0.48 | -0.91 |
| Peru | -0.61 | 0.13 | -1.34 |
| Philippines | -0.61 | 0.47 | -1.68 |
| Poland | -2.03 | -0.96 | -3.09 |
| Portugal | -1.69 | -0.58 | -2.78 |
| Puerto Rico | -0.01 | 1.26 | -1.27 |
| Qatar | -0.07 | 1.28 | -1.4 |
| Republic of Korea | -2.31 | -1.14 | -3.48 |
| Republic of Moldova | -0.51 | 0.48 | -1.48 |
| Romania | -0.52 | 0.22 | -1.25 |
| Russian Federation | 0.99 | 2.08 | -0.08 |
| Rwanda | -2.59 | -1.95 | -3.22 |
| Saint Kitts and Nevis | -2.74 | -2.09 | -3.38 |
| Saint Lucia | -1.69 | -0.98 | -2.4 |
| Saint Vincent and the Grenadines | -1.06 | -0.45 | -1.67 |
| Samoa | -0.12 | 0.7 | -0.93 |
| San Marino | 1.18 | 2.92 | -0.52 |
| Sao Tome and Principe | -0.06 | 0.55 | -0.67 |
| Saudi Arabia | 1.54 | 3.54 | -0.42 |
| Senegal | 0.01 | 0.77 | -0.75 |
| Serbia | -0.85 | -0.04 | -1.66 |
| **Countries and territories** | **EAPC**^1^ **(%)** | **Upper** | **Lower** |
| Seychelles | -0.55 | 0.14 | -1.24 |
| Sierra Leone | 1.06 | 1.81 | 0.32 |
| Singapore | -3.47 | -2.28 | -4.64 |
| Slovakia | -0.18 | 0.81 | -1.16 |
| Slovenia | -2.06 | -0.92 | -3.18 |
| Solomon Islands | 0.15 | 0.7 | -0.4 |
| Somalia | -0.25 | 0.38 | -0.87 |
| South Africa | -0.04 | 0.68 | -0.76 |
| South Sudan | -0.74 | 0.05 | -1.52 |
| Spain | -0.63 | 0.72 | -1.97 |
| Sri Lanka | 0.17 | 1.63 | -1.28 |
| Sudan | -0.67 | 1.05 | -2.35 |
| Suriname | -0.46 | 0.27 | -1.18 |
| Sweden | -1.11 | 0.34 | -2.54 |
| Switzerland | -2.24 | -0.74 | -3.71 |
| Syrian Arab Republic | -0.76 | 1.48 | -2.95 |
| Taiwan (Province of China) | -3.63 | -2.72 | -4.53 |
| Tajikistan | -2.05 | -0.69 | -3.4 |
| Thailand | -2.36 | -1.51 | -3.21 |
| Timor-Leste | -0.81 | 0.24 | -1.84 |
| Togo | -0.41 | 0.31 | -1.14 |
| Tokelau | -0.68 | 0.01 | -1.36 |
| Tonga | -0.73 | -0.02 | -1.44 |
| Trinidad and Tobago | -1.4 | -0.61 | -2.19 |
| Tunisia | -0.46 | 1.21 | -2.11 |
| Turkey | -1.42 | 0.38 | -3.18 |
| Turkmenistan | 1.05 | 2.14 | -0.04 |
| Tuvalu | -0.82 | -0.13 | -1.51 |
| Uganda | 0.1 | 0.77 | -0.56 |
| Ukraine | -2.31 | -1.27 | -3.34 |
| United Arab Emirates | -1.19 | -0.17 | -2.19 |
| United Kingdom | -1.82 | -0.49 | -3.12 |
| United Republic of Tanzania | -0.35 | 0.34 | -1.04 |
| United States Virgin Islands | -1.16 | -0.2 | -2.11 |
| United States of America | -0.69 | 0.66 | -2.03 |
| Uruguay | -0.47 | 0.38 | -1.31 |
| Uzbekistan | 0.15 | 1.13 | -0.82 |
| Vanuatu | -0.24 | 0.53 | -1 |
| Venezuela (Bolivarian Republic of) | -0.31 | 0.4 | -1.02 |
| Viet Nam | 0.03 | 1.01 | -0.94 |
| Yemen | -0.29 | 1.35 | -1.91 |
| Zambia | -1.03 | -0.45 | -1.61 |
| **Countries and territories** | **EAPC**^1^ **(%)** | **Upper** | **Lower** |
| Zimbabwe | 0.83 | 1.42 | 0.23 |

^1^ EAPC, estimated annual percent change

# References

[1] World Health Organization (WHO), Sexual and reproductive health, (n.d.). https://www.who.int/reproductivehealth/publications/cancers/cervical-cancer-guide/en/.

[2] L. Bruni, M. Diaz, L. Barrionuevo-Rosas, R. Herrero, F. Bray, F.X. Bosch, S. de Sanjosé, X. Castellsagué, Global estimates of human papillomavirus vaccination coverage by region and income level: A pooled analysis, Lancet Glob. Heal. 4 (2016) e453–e463. https://doi.org/10.1016/S2214-109X(16)30099-7.

[3] M. Poljak, K. Seme, P.J. Maver, B.J. Kocjan, K.S. Cuschieri, S.I. Rogovskaya, M. Arbyn, S. Syrjänen, Human papillomavirus prevalence and type-distribution, cervical cancer screening practices and current status of vaccination implementation in Central and Eastern Europe., Vaccine. 31 Suppl 7 (2013) H59-70. https://doi.org/10.1016/j.vaccine.2013.03.029.

[4] S. Portet, A primer on model selection using the Akaike Information Criterion., Infect. Dis. Model. 5 (2020) 111–128. https://doi.org/10.1016/j.idm.2019.12.010.
